# Systematic review: bioethical implications for COVID-19 research in low prevalence countries, a distinctly different set of problems

**DOI:** 10.1186/s12910-021-00589-4

**Published:** 2021-03-03

**Authors:** Tony Skapetis, Constance Law, Rohan Rodricks

**Affiliations:** 1 Sydney Dental School, Faculty of Medicine and Health, University of Sydney, Westmead Campus, NSW 2145 Australia; 2 Division of Oral Health, Western Sydney Local Health District, Westmead, NSW Australia

**Keywords:** COVID-19, Ethics, Low-prevalence, Challenges, Research, Systematic review, Recommendations

## Abstract

**Background:**

The COVID-19 pandemic has presented extraordinary challenges to worldwide healthcare systems, however, prevalence remains low in some countries. While the challenges of conducting research in high-prevalence countries are well published, there is a paucity from low COVID-19 countries.

**Methods:**

A PRISMA guided systematic review was conducted using the databases Ovid-Medline, Embase, Scopus and Web of Science to identify relevant articles discussing ethical issues relating to research in low prevalence COVID-19 countries.

**Results:**

The search yielded 133 original articles of which only 2 fit the inclusion criteria and aim, with neither specific to low prevalence. Most of the available literature focused on clinical management and resource allocation related to high prevalence countries. These results will be discussed under the ethical dimensions of equity, individual liberty, privacy and confidentiality, proportionality, public protection, provision of care, reciprocity, stewardship and trust..

**Conclusions:**

A systematic review failed to identify articles relating to COVID-19 research ethics, specific to low prevalence countries. It shows that there is a significant gap in the literature that warrants further investigation. Common ethical principles were used to present a distinct set of challenges experienced by a country with a low prevalence of COVID-19. This unique perspective of some of the common ethical problems surrounding research, may help guide further discussion and guide research in similar countries.

## Background

The COVID-19 pandemic has presented an extraordinary challenge to worldwide healthcare systems, as well as clinicians and researchers. The virus initially began in Asia in November 2019 and spread across Europe, the United States and into Australia and other regions, affecting more than a million people worldwide within a few months. As global communities deal with the COVID-19 pandemic, many challenging legal, social and ethical issues have arisen. The World Health Organization (WHO) has outlined the ethical obligations of healthcare providers during a pandemic under three distinct categories: moral, professional and legal [1].

The National Institute for Health [2] has proposed seven guiding principles to guide the conduct for ethical research: social and clinical value, scientific validity, fair subject selection, favorable risk–benefit ratio, independent review, informed consent and respect for subjects. These are similar to the values set out in the National Statement on Ethical Conduct in Human Research in Australia [3]. Values such as respect for participants, research merit and integrity, justice and beneficence help to shape the relationship between researchers and subjects as one of trust, mutual responsibility and ethical equality.

Respect recognizes the value of the individual and their autonomy in the control of their life and decisions. Justice involves a regard for human sameness that each person shares with every other. This translates to an equal and fair distribution of benefits and burdens of the research and in a fair and equitable treatment in recruitment of participants and research reviews. Beneficence in research involves assessing the risk of harm and potential benefits of research to the participants and to the wider community. Additionally, it considers the welfare and interests of the researchers and associated people while reflecting on the social and cultural implications of their work.

Measures of COVID-19 prevalence including what constitutes low or high prevalence may be problematic given differences in diagnostic approaches, time intervals being considered and variations in sampling methodology [4]. As such, this paper has utilized a pragmatist interpretation of what would be accepted as a country representing low prevalence.

In countries identified as having a low prevalence of COVID-19 such as Australia (www.covid19data.com.au), the available pool of research subjects is considerably reduced. Conversely, due to the pandemic, the research needs have increased. This adds to the potential burdens placed on affected individuals to participate in COVID-19 related research. Ethics committees are usually tasked with approving research based on ethical principles and benefits to the individual and/or community. It follows that there may be a distinctly different set of ethical challenges involved with conducting research in low COVID-19 prevalence countries. This highlighted a necessity to carry out a systematic review of available literature to help frame these research challenges.

## Aim

To determine the ethical challenges associated with conducting medical research in low COVID-19 prevalence countries.

## Methods

### Search strategy for articles

Eligible original articles published between 2000 and 2020 were identified using the following search terms nomenclature, (ethic$ or bioethic$) and COVID-19 and (considerations or recommendations or challenges or framework), across the following databases: 1. OVID-Medline, 2. Embase, 3. Scopus, and 4. Web of Science. The search strategy was first run in consultation with a qualified hospital librarian, followed by independent searches by two of the authors. The initial search results were compared, any differences in the search results were then discussed among the three authors to reach consensus.

### Screening of articles

A full-text assessment of the yield articles was conducted against the inclusion criteria. The articles deemed suitable were then grouped and further reviewed independently by all three authors for inclusion or exclusion. Differences were then resolved by re-reading and discussion until consensus was reached.

### Inclusion and exclusion criteria

Articles that were published in English, contained abstracts, limited to humans and discussed ethical considerations pertaining to COVID-19 research were included. Exclusion criteria and the number of articles excluded are summarized in Table 1.

**Table 1:** Exclusion criteria and number of articles excluded

Exclusion criteria	No. of excluded articles
1. Non-ethics-related COVID-19 studies	7
2. Clinical management studies	44
3. Resource allocation studies	34
4. Case studies	6
5. Non-English publications	9
6. Application of technologies	8
7. Staff health	5
Total	113

## Results

**Fig. 1:**
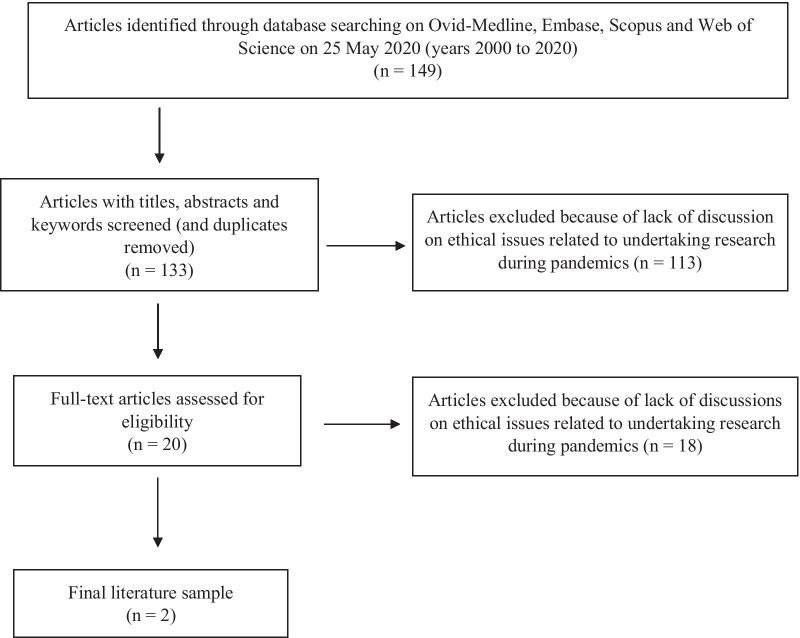
PRISMA diagram of the literature search results

The search results are shown in the PRISMA (http://www.prisma-statement.org/) diagram, Fig. 1 and a PRISMA checklist is included in the supplementary files (Additional file 1). A total of 133 abstracts were identified. One hundred and thirteen were excluded, based on abstracts, because they did not meet the inclusion criteria. The complete articles of the remaining 20 studies were further evaluated with eighteen additional articles excluded as they did not adequately address the aim of the study.

### Excluded articles

The systematic review initially excluded 113 articles with regards to each exclusion criteria shown in Table 1. A further 18 articles were subsequently excluded, based on the nature of the article i.e. editorial/commentary, or lacking relevance to the inclusion criteria.

### Included articles

A total of 2 articles were included in the final analysis (Table 2).

**Table 2:** Summary of the two articles included in the study

Authors	Summary
Padala et al. [5]	This article discussed the application of ethical principles decision-making process from a research perspective. It suggested that majority of research participants believed that it is important to continue medical research during epidemics. The authors discussed how local and national guidance, staffing strain, institutional support, participants’ perception on research participation affect the decision-making process and outline potential changes needed and guideline for regulatory bodies
Kramer et al. [6]	The article examined ethical issues during the HIV/AIDS pandemic and draws comparisons to the existing COVID-19 pandemic. The authors provided ethical analysis and recommendations regarding professional responsibilities, patient’s right, resource allocation and research-related issues. The article also discussed the ethical issues around end-of-life decisions

## Discussion

Only Padala [5] and Kramer [6] fit the inclusion criteria but neither author discussed ethical considerations within low prevalence countries. Furthermore, only Padala et al. considered research specific ethical issues, although they were only applicable to high prevalence societies.

One of the two final articles [6] reflected on ethical issues during the HIV/AIDS pandemic which were used to navigate ethical dilemmas associated with the COVID-19 pandemic. The theme of privacy and confidentiality included the balance of disclosure of patient COVID-19 status against the protection of healthcare workers. Provision of care was discussed in terms of healthcare workers treatment responsibilities and access to personal protective equipment (PPE). The ethical issues around equity were considered for resource allocation and individual liberty in terms of decisions about end of life.

The 2^nd^ included article, by Padala, provided evidence that 82% of study participants believed it important to continue conducting medical research during epidemics. The manuscript examined the ethical issues surrounding COVID-19 research from both the perspective of the investigator and participants (n = 51). Visit, policy related factors and workforce issues were related to the ethical constructs of stewardship and provision of care while participant perspectives and patient visits were discussed in terms of trust and protection of the public.

Considering the paucity of articles specific to the research question, a relevant COVID-19 ethical framework [7], arising from the literature search, was employed. This framework uses 9 ethical dimensions and was used as a scaffold to help describe a series of unique ethical problems associated with conducting research in a low COVID-19 prevalence environment (Table 3).

**Table 3:** Ethical dimensions and problems

Ethical dimensions	Associated problems
Equity	International research collaborations to include/inclusion of low prevalence countries become less attractive
Individual liberty	Patient’s liberty to choose which study to participate when there are multiple studies concurrently
Privacy and confidentiality	The risk of identification
Proportionality	Disproportionate cessation and continuation rules for other research projects
Public protection	Research being re-directed outside hospital protective frameworks and into communities and care facilities with greater COVID numbers Movement restrictions may hinder research participation
Provisional of care	Patients may be subjected to oversampling when the sample size is small
Reciprocity	Difficult to align and maximize skills of redeployed personnel Diminished perception of self-benefit among health staff
Stewardship	Research may be more scrutinized and be harder to substantiate in terms of public good
Trust	Forced sharing of samples by competing researchers and forced collaborations amongst competitor research groups

### Equity

#### Problem: international research collaborations to include Australia & initiated from Australia become less attractive

Low prevalence countries may be disadvantaged by not being included in more global COVID-19 vaccine research and other related clinical trials. Additionally, such countries may not readily be offered research funding from international charitable foundations [8]. and pharmaceutical sponsors. This is made worse if the low prevalence is associated with higher research ethical and governance standards that may be seen to hinder research [9].

In contrast, the experience from high prevalence countries, many of which are also low and middle income countries with looser regulation, is a population more eager to participate in COVID-19 research as a way of accessing often unaffordable health care [10]. The same author further describes the disproportionate burdens the pandemic has placed on elderly residents in nursing homes without a corresponding equitable increase in research to improve health outcomes among this cohort. This observation around the need for more equitable access to both the benefits and burdens of research is equally relevant to both high and low COVID prevalence countries [11].

### Individual liberty

#### Problem: from the patient’s perspective, how do patients choose which study to consent to when being faced with many studies concurrently?

The rights of the individual and the obtaining of consent are paramount in any research involving health systems. Furthermore, each patient should be at liberty, given adequate information, to assess their own perceptions of participatory benefit versus burden which may be physical, psychological, economic, familial and social [12].

The flip side to this, is a construct supported by some researchers that proposes that all persons should participate in biomedical research unless they have a good reason not to. This is premised on the concept that research leads to a better and longer lived society [13].

In low prevalence countries, few COVID affected patients may be subjected to an overwhelming number of requests for study participation, with decision making becoming difficult. This concept of respondent burden according to Ulrich et al., is poorly researched and varies in intensity and degree depending on the patient’s perception of risk, the subject’s condition, prognosis, mental state and available support [14].

The situation is quite different however, in countries with larger populations affected by COVID-19, where the burden and possible perceived obligation of research participation is well diluted and more equitably distributed among the population.

### Privacy & confidentiality of individuals

#### Problem: the risk of identification e.g. data fields such as sex and age may be identifiable

In countries with a large prevalence of COVID-19 and thousands of patients involved, it becomes more difficult to identify individuals who have contacted the disease and furthermore been hospitalized. Typical essential demographic data such as age and gender, often collected as part of clinical research, would normally not allow identification of participants outside of hospital record boundaries. In a low prevalence country, however, supported by a liberal and well informed media, the situation may arise where it is heralded that there may be only a handful of COVID-19 positive inpatients within a large hospital of which only one is a middle aged male. In such cases, maintaining patient confidentiality becomes problematic. Additionally, the low prevalence may attach a disproportionate risk of stigmatism attached to research participation [15] which is likely more diluted in countries with higher prevalence.

### Proportionality

#### Problem: disproportionate cessation and continuation rules

The National Health and Medical Research Council in Australia produced a national guidance document in March 2020, to assist institutions, HREC’s, researchers and sponsors regarding research activity during COVID-19 [7]. Despite the low prevalence in Australia, this document could subsequently be interpreted differently by various jurisdictions for imposing varying contingencies and restrictions upon research activities. One such interpretation involved the complete suspension of any new research other than COVID-19 related, for several months within NSW, Australia, during the initial period when the prevalence was uncertain. The difference between the perceived and actual COVID-risk has negatively impacted on the approval of new research projects. This has left many patients stranded from inclusion into potentially life-saving clinical trials. This conundrum has been mirrored however in high prevalence countries creating similar ethical concerns around the proportionality of the response relative to the perceived CORONA threat [16].

### Protection of the public

#### Problem: research being re-directed outside hospital protective frameworks and into communities and care facilities with greater COVID numbers

With the lower number of COVID presentations requiring hospital admission, researchers in low prevalence countries have had to shift their recruitment protocols to be more community focused away from the greater protections provided by the hospital environment. This may present its own set of ethical issues. For example, hospital based research would normally be well supported in terms of facilitated recruitment, from a substantial staffing and resource perspective. While on the other hand, Strike et al. [17] describes how non-hospital research may be more negatively affected in terms of recruitment and protocol compliance, where appointments are easily forgotten and the research is more reliant on unsupervised outpatient participation. Additionally, the same author noted greater confidentiality issues associated with community based research as it could impact on participants personal relationships when being conducted within smaller care facilities as would be the case for smaller nursing homes.

#### Problem: strong movement restrictions hinder research participation

Low prevalence countries have attributed their success against COVID-19, at least in part, to a prioritization in favor of the preservation of lives ahead of economic losses [18]. This has been largely achieved through early and almost draconian restrictions to people’s freedom of movement exemplified by the constant message to “stay at home” from the Australian prime minister.

Beyond the well accepted harmful consequences such as increased unemployment, the rise in domestic violence, as well as the increase in depression and anxiety [18], far reaching restrictions limiting people’s movements, has also negatively impacted clinical research. Participants are unable to attend research related appointments to receive study medications, treatment and testing thereby possibly compromising study results. Additionally, the restrictions make it more difficult for the researchers themselves to visit sites for site initiation, monitoring, auditing and for problem solving purposes, thereby diminishing their obligations to both patient and study sponsors.

### Provision of care

#### Problem: subjecting COVID-19 patients to oversampling (oversampling)

There have been several guidelines published for maximum blood draw for participants in research addressing children including those by the European Union [19], Peplow et al. [20] and the Mayo Clinic [21] as well as for adults including from the United States National Institute of Health [22] and Oregon State University [23].

Despite these international guidelines, there appeared to be no similar agreed or endorsed guidelines for Australia. Given the low numbers of COVID-19 positive patients in Australia and the large influx of clinical studies wishing to obtain blood samples, a large Australian public local health district responded by setting up a ‘COVID-Connect’ group. This group was widely represented by a diversity of research stakeholders including local health district executives, research institutes, universities, pathology services, government research entities, specialist clinicians and bioethicists. The role of this committee was to connect and navigate all COVID related studies and prioritize them in terms of scientific validity, potential clashes with existing and proposed research, alignment with the local health district priorities and the degree of impact on the few COVID patients. One of the deliberations of this COVID-Connect group was to draft recommendations on maximum research blood draw levels for the local health district hospitals. At the time of the literature search, across either the included or excluded papers, there did not appear to be any equivalence elsewhere to COVID-Connect.

### Reciprocity

#### Problem: difficult to align and maximize skills of redeployed personnel.

Redeployment of staff has been a widely used strategy to help manage the surge of COVID during the pandemic [24]. Common themes in literature from high COVID countries when dealing with the concept of reciprocity among deployed health clinicians, surrounds the concept of increased clinician risk without a parallel increase in compensation and consideration [24, 25]. Other authors [26, 27] raise the possibility of medical negligence in terms of deployed clinicians delivering a lower standard of care compared to pre-COVID.

In low prevalence countries, considerable redeployment may similarly occur in anticipation for COVID-19 cases far beyond what is realized. In parallel, many allied health services, such as dental, may be curtailed in order to reduce disease spread and conserve PPE thereby providing large numbers of surplus staff requiring redeployment. This may create a large surplus of highly qualified clinicians that cannot be utilized to their full capabilities and who are redeployed to less clinically relevant areas such as corporate, cleaning, maintenance and administrative services. During redeployment of health care workers, a lack of adequate training and difficulties in matching skillsets to the areas of redeployment, have highlighted the importance of conducting detailed skills assessments [28]. This redeployment misalignment may contribute to lower clinician self-esteem and self-worth in terms of perceived contribution to the pandemic. Dunham describes how pandemics may challenge clinician’s morals with competing obligations manifesting as moral distress.

#### Problem—With lower prevalence there is a diminished perception of self-benefit among health staff

The pandemic has seen an influx of COVID related research involving healthcare workers [29]. The drivers behind having these workers participate in voluntary research within low prevalence countries may be different. Firstly, the direct impact COVID-19 has placed on these workers is considerably diminished compared to experiences in high prevalence countries where sick and dying colleagues and relatives would be expected. Additionally, given the lower risk exposure to COVID-19 in low prevalence settings, health workers may feel that the inherent risks posed through research participation may be disproportionate to the perceived gains. This hesitancy with healthcare workers involvement with any potential COVID-19 vaccines has been reported as being related to risk–benefit perceptions, safety concerns as well as perceived infection risk [30].

### Stewardship

#### Problem: research may be more scrutinized and be harder to substantiate in terms of public good

With low prevalence of COVID-19 positive cases, the earlier mentioned COVID-Connect group was commissioned to provide advice relating to COVID research in terms of scientific integrity and benefit. This was because it was difficult to support all research activities, despite ethics approval, many of which were investigator initiated with minimal budgets and heavy reliance on the goodwill support of only a few departments such as the intensive care and COVID wards of the local health district.

Closely aligned is also the inherent difficulty in achieving sample size and attracting any additional research funding as well as in-kind support by the health districts for studies which may have to be extended. Additionally, benefits of lower prevalence also come with a disproportionately greater public and media scrutiny on fewer clinical studies especially when there is a lack of success for example in achieving adequate recruitment and a failure to demonstrate efficacy or safety [31].

### Trust

#### Problem: forced sharing of samples by competing researchers and forced collaborations amongst competitor research groups

COVID 19 has seen a massive influx of related publications, for example, a manual search using Publons of journal papers and preprints added on the 19th of June gave a result of 124 papers for a single day (www.publons.com). Most of these papers include multiple authors from various institutions and it has been reported that research collaboration promotes research productivity and that research productivity positively influences collaborations both intra and intermural [32]. This same study interestingly found that domestic collaborations improved research productivity more.

The experience with low COVID-19 prevalence, has seen an imperative for more researcher collaboration despite inherent competitiveness from different research groups and specialties. An example has been the sharing of clinical samples from few affected patients. Researchers after overcoming issues of trust have combined blood draws to minimize patient discomfort and improve efficiency while at the same time preserving PPE. Similarly, qualitative researchers with similar methodologies have considered working together and hybridizing their studies thus making them more likely to gain support from the COVID-Connect group and local health district.

#### Limitations

The authors wish to acknowledge the limitations of this systematic review. Firstly, much of the literature surrounding the topic of ethics relating to research, presents itself in qualitative terms and often consists of narrative and argument represented through philosophical interpretations. It is therefore difficult to apply concepts such as study bias, study design and synthesis of results, in a meaningful way [33]. Secondly, the research aim itself may not have adequate focus and lend itself to interpretational errors. None the less, the systematic methodology was used more in terms of a compass rather than an anchor to help substantiate the literature gap as well as help aggregate common ethical themes surrounding COVID-19. Furthermore, this study is limited by the databases used, and that it only included publications in English. As the COVID-19 pandemic is rapidly evolving articles published after the search date would not be included in this study.

## Conclusion

The COVID-19 pandemic, poses challenges to researchers in high prevalence countries world-wide, with similarities being experienced in terms of prevailing ethical problems. Most of the available literature identified using systematic review methodology, focuses on clinical management and resource allocation, particularly in areas with high prevalence. There is a paucity of publications relating to countries with low COVID-19 prevalence. This review used prevailing ethical principles to present a distinct set of challenges experienced by a country with a low prevalence of COVID-19. This unique perspective of some common ethical problems surrounding clinical research, may help provide insights, guide further discussion and assist research in similar countries.

## Supplementary information

**Additional file 1:** PRISMA checklist.

## Data Availability

The dataset consisting of search results, including details of excluded papers, are available from the corresponding author on reasonable request.

## Abbreviations

COVID-19: Coronavirus disease of 2019
PRISMA: Preferred reporting items for systematic reviews and meta-analyses
WHO: World Health Organisation
HIV/AIDS: Human immunodeficiency virus/ acquired immunodeficiency syndrome
PPE: Personal protective equipment
NSW: New South Wales
HREC: Human research and ethics committee
